# Identification of hybrids between the Japanese giant salamander (*Andrias japonicus*) and Chinese giant salamander (*Andrias* cf. *davidianus*) using deep learning and smartphone images

**DOI:** 10.1002/ece3.10698

**Published:** 2023-11-09

**Authors:** Kosuke Takaya, Yuki Taguchi, Takeshi Ise

**Affiliations:** ^1^ Graduate School of Agriculture Kyoto University Kyoto Japan; ^2^ Hiroshima City Asa Zoological Park Hiroshima Japan; ^3^ Field Science Education and Research Center Kyoto University Kyoto Japan

**Keywords:** amphibian, biological invasions, EfficientNetV2, gradient‐weighted class activation mapping, hybridization

## Abstract

Human‐mediated hybridization between native and non‐native species is causing biodiversity loss worldwide. Hybridization has contributed to the extinction of many species through direct and indirect processes such as loss of reproductive opportunity and genetic introgression. Therefore, it is essential to manage hybrids to conserve biodiversity. However, specialized knowledge is required to identify the target species based on visual characteristics when two species have similar features. Although image recognition technology can be a powerful tool for identifying hybrids, studies have yet to utilize deep learning approaches. Hence, this study aimed to identify hybrids between the native Japanese giant salamander (*Andrias japonicus*) and the non‐native Chinese giant salamander (*Andrias* cf. *davidianus*) using EfficientNetV2 and smartphone images. We used smartphone images of 11 individuals of native *A. japonicus* (five training and six test images) and 20 individuals of hybrids between *A. japonicus* and *A.* cf. *davidianus* (five training and 15 test images). In our experimental environment, an AI model constructed with EfficientNetV2 exhibited 100% accuracy in identifying hybrids. In addition, gradient‐weighted class activation mapping revealed that the AI model was able to classify *A. japonicus* and hybrids between *A. japonicus* and *A.* cf. *davidianus* on the basis of the dorsal head spot patterning. Our approach thus enables the identification of hybrids against *A. japonicus*, which was previously considered difficult by non‐experts. Furthermore, since this study achieved reliable identification using smartphone images, it is expected to be applied to a wide range of citizen science projects.

## Graphical abstract

Management of hybrids is essential for biodiversity conservation; however, the distinction between the two species becomes a challenge in cases of hybrids with similar characteristics to native species. Hence, we identified hybrids between native Japanese giant salamanders (*Andrias japonicus*) and non‐native Chinese giant salamanders (*Andrias* cf. *davidianus*) using deep learning and smartphone images.

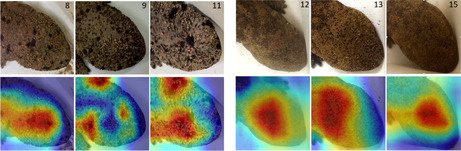

## 1 INTRODUCTION

Although significant effort has been devoted toward conservation, biodiversity loss remains a global challenge (Johnson et al., 2017). Anthropogenic activities such as urbanization, agricultural intensification, and species exploitation reduce biodiversity, and species extinction rates are progressing much faster than in the past (Ceballos et al., 2015). In addition, globalization has led to the introduction of organisms into new environments, establishing non‐native populations in new areas (Pyšek et al., 2020). These non‐native species negatively affect the ecosystem through direct and indirect effects such as predation, niche displacement, and introduction of diseases (Doherty et al., 2016; Haubrock et al., 2021; Kortz & Magurran, 2019; Scheele et al., 2019). Moreover, non‐native species are recognized as a further driver of the extinction of local species (Bellard et al., 2016). Therefore, the mitigation of biological invasions is essential to conserve biodiversity because the impact of non‐native species on biodiversity and ecosystems is expected to increase in the future (Pyšek et al., 2020).

When non‐native species are introduced into a new habitat, they sometimes encounter close relatives. In such cases, hybridization occurs owing to incomplete reproductive isolation from closely related species (Todesco et al., 2016). Hybridization in non‐native species is frequently observed and considered an evolutionary mechanism that determines invasion success (Bock et al., 2021). For example, native California tiger salamanders (*Ambystoma californiense*) and introduced barred tiger salamanders (*Ambystoma tigrinum mavortium*) have hybridized and formed a hybrid swarm in California. Fitzpatrick and Shaffer (2007) reported that hybrid tiger salamanders exhibited higher fitness than individuals containing mostly native or mostly introduced alleles (hybrid vigor). Hybrid vigor is defined as the superior growth or reproduction of hybrids compared with parental lineages (Vilà & D'Antonio, 1998); this genetic admixture can increase the fitness of colonizers in biological invasion (Qiao et al., 2019). In addition, hybrids sometimes have intermediate traits or different traits from the parent species (Hayden et al., 2011), and some traits may determine the establishment success of non‐native species (Coulter et al., 2020). For instance, a meta‐analysis of plants, animals, and fungi demonstrated that non‐native hybrids have a larger body size and are more fecund than their parent species (Hovick & Whitney, 2014). Although early non‐native populations are affected by density‐dependent processes such as the Allee effect (Camacho‐Cervantes et al., 2023), hybridization provides mating partners for non‐native species, which could reduce the Allee effect and promote invasions (Yamaguchi et al., 2019).

Hybrids of similar species pose a threat to genetic diversity because introduced alleles may eventually replace the native alleles (Fitzpatrick & Shaffer, 2007). Although it is necessary to control hybrids to conserve biodiversity, the difficulty in distinguishing between native and hybrid species is one of the critical issues in managing and controlling hybrids. Hybrids can often be detected using morphological characteristics (Allendorf et al., 2001). However, morphological characteristics cannot be used to determine whether an individual is a first‐generation or backcross‐generation hybrid. In addition, the misidentification of species can also cause conservation problems. For example, incorrect identification of target species could negatively impact native species; native frogs have been killed in Australia because of misjudgments while removing the non‐native cane toad (*Rhinella marina*) (Somaweera et al., 2010).

The development of molecular genetic techniques, such as PCR and eDNA, has overcome these challenges (Allendorf et al., 2001; Rees et al., 2017). DNA analysis allows accurate species identification and can reveal the degree of hybridization, previously difficult to determine using morphological traits. However, the cost of molecular analysis remains high for some methods, and laboratory work and expertise are required to analyze samples (Martinez et al., 2020; Stein et al., 2014). On the contrary, visual identification of target species using photographs is less expensive, and data can be easily collected with minimal disturbance for the individuals (Hou et al., 2020). In addition, citizen science surveys using photographs are a valuable approach for the early detection of non‐native species because they can be used to collect data over large areas (Werenkraut et al., 2020). For example, new tools and datasets such as iNaturalist and eBird are emerging that allow people to report observations at any time and from any location (Larson et al., 2020). Despite these advantages, photographic identification is time‐consuming when the observer must check large databases (Bogucki et al., 2019).

In recent years, deep learning image recognition technology, a novel group of artificial intelligence approaches, has begun to be utilized to identify both species and individuals in ecology. Identifying and counting animal species in images provides basic but essential information (Tuia et al., 2022). Many previous studies have combined camera traps and deep learning to identify species. For instance, Norouzzadeh et al. (2018) identified wild mammals and birds using 3.2 million images obtained from camera traps in the Serengeti National Park. In addition, these techniques have been applied to individual identification, such as green turtles (Carter et al., 2014), chimpanzees (Schofield et al., 2019), and brown bears (Clapham et al., 2020). Furthermore, deep learning algorithms have already been used to detect non‐native species. For example, Ashqar and Abu‐Naser (2019) classified *Hydrangea* with a dataset containing approximately 3800 images taken in a Brazilian national forest, demonstrating the feasibility of this approach. Guo et al. (2022) also developed a novel deep learning model to identify common reed (*Phragmites australis*) from unmanned aerial vehicle (UAV) images. In another study, tall goldenrod (*Solidago altissima*) was detected from action camera images using the chopped picture method, and the suitability of this method in citizen science was considered (Takaya et al., 2022). Although a similar approach may provide a new method for identifying hybrids, studies have yet to apply deep learning models to their identification.

Deep learning has achieved remarkable success in various fields, although its lack of transparency is a major disadvantage (Kakogeorgiou & Karantzalos, 2021; Petch et al., 2022). This technique is sometimes considered a “black box” method in that it is unclear how and why a particular classification decision is arrived at (Brunese et al., 2020; Montavon et al., 2017). Recently, several approaches have been developed to overcome this challenge. For example, gradient‐weighted class activation mapping (Grad‐CAM) provides a heatmap visualization of the regions that influenced the model's predictions, giving valuable information for the interpretation of results (Selvaraju et al., 2017). In ecological research, Grad‐CAM is applied in individual re‐identification (De Silva et al., 2022) and species identification (Banan et al., 2020). Although this technique provides visual information for classifying hybrids, research applying this technique to detect hybrids in biological invasions is lacking.

**FIGURE 1:**
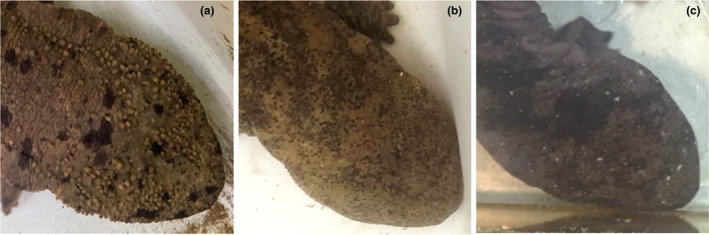
Dorsal head spot patterning of *Andrias japonicus* (a), HYB (b), and *Andrias davidianus* (c). The image of *A. davidianus* was provided by Dr. Benjamin Tapley, Zoological Society of London.

The Japanese giant salamander (*Andrias japonicus*) is an amphibian endemic to Japan and is threatened with extinction as a result of decreasing population due to habitat degradation and fragmentation (Taguchi & Natuhara, 2009; Tochimoto et al., 2007; Yamasaki et al., 2013). In the 2022 IUCN Red List, the conservation status rank of this species was changed from Near Threatened to Vulnerable (IUCN, 2022). One reason for the status change in *A. japonicus* is the hybridization with the congeneric but non‐native Chinese giant salamander (*Andrias* cf. *davidianus*). This species is also threatened with extinction in its original habitat, but individuals introduced to Japan in the early 1970s have become wild and hybridized with *A. japonicus*. For example, a Kyoto City government survey revealed that only four (2%) out of 244 individuals captured in the Kamo River Basin in Kyoto were native *A. japonicus*, and the remaining 240 (98%) were *A*. cf. *davidianus* or hybrids between *A. japonicus* and *A*. cf. *davidianus* (HYB), a problem requiring rapid action (The Kyoto City Government, 2015). Moreover, the number of areas where HYB have been caught is increasing, with hybrids already confirmed in eight prefectures in western Japan (Kyoto, Mie, Nara, Shiga, Okayama, Hiroshima, Aichi, and Gifu). Currently, HYB is identified by visual screening and DNA analysis (Fukumoto et al., 2015). Although detecting HYB by spot patterning would allow their rapid identification in the field, this approach requires specialized knowledge (Figure 1). Generally, *A. davidianus* has a darker body color with paler spots than *A. japonicus*, although the body color and spot patterning differ among individuals of both species. The accurate identification of HYB from images would require less time and expense than DNA analysis. It would also facilitate the early detection and effective capture of suspected HYB individuals via citizen science, particularly in areas where hybrids have not yet been found, thereby contributing to the effective conservation of *A. japonicus*.

Our aim was to identify HYB using a computer‐based algorithm employing deep learning. The wide availability of the Internet and smartphones provides the opportunity for identifying species and reporting their locations (Larson et al., 2020). Our approach allows the public to photograph and detect HYB individuals without specialized knowledge because *A. japonicus* and HYB often appear in rivers flowing through urban areas and less populated rural areas. In recent years, citizen science has been adopted to manage non‐native species (Larson et al., 2020), and a similar method could be applied to HYB. Secondly, we developed an efficient method to recognize *A. japonicus* and HYB. Spot patterns are more difficult to quantify than morphological traits such as body size; thus, few people can utilize this information. However, techniques such as Grad‐CAM allow visualization of the important region for predicting whether the species is *A. japonicus* or HYB by the AI model. If specific essential areas for identifying HYB can be clarified, that information will be valuable for helping the general public to identify HYB.

## 2 MATERIALS AND METHODS

### 2.1 Image acquisition

In this study, 11 individuals of native *A. japonicus* and 20 individuals of HYB were used (Figures S1 and S2). The Chinese giant salamander has been categorized into several species in recent years (Chai et al., 2022; Turvey et al., 2019; Yan et al., 2018). Because it is unknown which Chinese *Andria*s species was introduced to Japan, we will be referred to as *Andrias* cf. *davidianus* in this study. The native individuals were kept at the Conservation Breeding Facility in Hiroshima City Asa Zoological Park, which is a leading facility in Japan for the research, conservation, and breeding of this species. The HYB were captured in the wild and then transferred to this facility.

The 11 *A. japonicus* individuals were photographed on August 20, 2022, at 11:00 a.m. Each individual in the water was recorded on Full HD video from above (Figure S3) for approximately 30 seconds using an iPhone 11, from which still images were obtained for analysis. The dorsal head spot patterning of *A. japonicus* was recorded at approximately 60 cm from the camera, and the water depth was about 20 cm. To reduce glare due to reflection from the water surface, the recording was performed under a black umbrella. The videos were converted to 10 still JPEG images (1920 × 1080) per second using Free Video to JPG Converter version 5.0.101 (DVDVideoSoft Ltd.).

The 20 HYB were recorded on Full HD video on November 19, 2022, at 2:00 p.m. using an iPhone SE 2020. The method of image collection was the same as that for *A. japonicus*.

### 2.2 Ethics declaration

*A. japonicus* are protected as a National Natural Monument under the Law for the Protection of Cultural Properties. Therefore, this study required permission and was approved by Hiroshima City Asa Zoological Park under the auspices of the Agency for Cultural Affairs and was categorized as a non‐invasive study.

### 2.3 Framework

**FIGURE 2:**
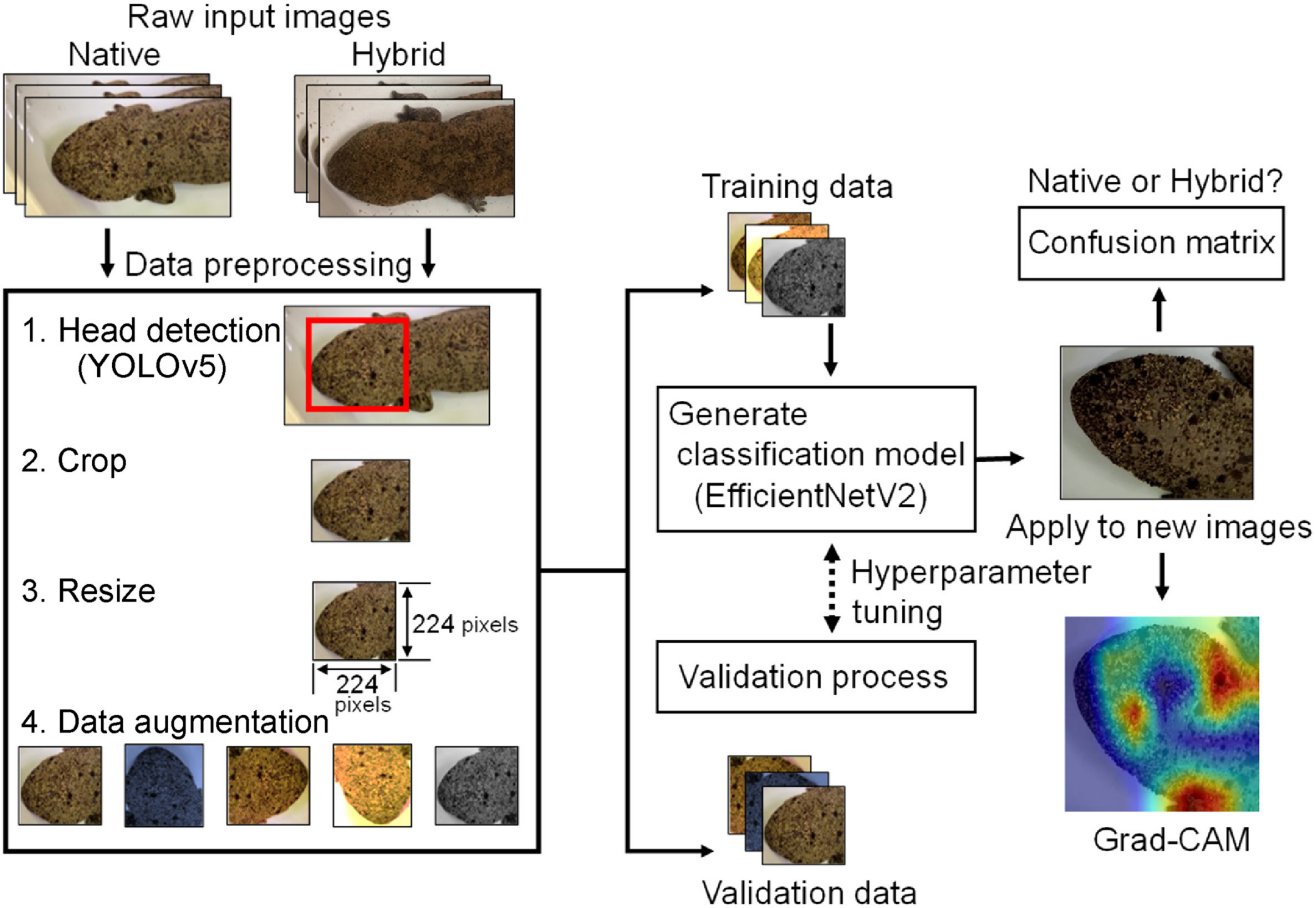
Framework of the classification model for *Andrias japonicus* and HYB using smartphone photographs.

The heads in the images were automatically detected using YOLOv5 (Redmon et al., 2016) and used as either training or test images (Figure 2). The training data comprised five individuals of *A. japonicus* and five HYB individuals selected randomly from the two groups (Table 1; Figures S1 and S2). The six remaining *A. japonicus* individuals and 15 remaining HYB individuals not used in training were selected as test images. These images were resized to 224 × 224 pixels to ensure consistency in size. Additionally, augmentation (rotation, crop, brightness, Gaussian noise, color jitter, and saturation) was applied to the training data to prevent overfitting. Each type of augmentation was applied with a probability of 50%. For example, applying rotation and cropping resulted in three patterns of images with (1) both processes applied, (2) one of the two processes applied, and (3) neither process applied. After augmentation, 70% of the images used for training and 30% of the images used for validation were randomly separated for analysis.

**TABLE 1:** Dataset summary showing the date and purpose of the images taken.

Species	Year	Date	Training	Test
*A. japonicus*	2022	August 20	468	525
Hybrids	2022	November 19	439	1356

### 2.4 Visualization using Grad‐CAM

Gradient‐weighted class activation mapping generates a heatmap that indicates the importance of pixels in the feature maps of an input image (Selvaraju et al., 2017). These highlighted regions in an image provide an explainable view of deep learning models. Using this method, we extracted the feature maps of the final convolutional layer in the model and calculated the gradients. These gradients were subjected to global average pooling to obtain the weights. We used a weighted combination of feature maps to form output images using the ReLU (rectified linear unit) function, which allows features with a positive effect on the category of interest to be identified.

### 2.5 EfficientNetV2

In this study, we trained EfficientNetV2 to classify images. This convolutional neural network scales down the number of layers while scaling down the model (Tan & Le, 2019). EfficientNetV2 is an improved version of EfficientNet with increased training speed and parameter efficiency (Tan & Le, 2021). The EfficientNetV2 model employs a neural architecture search (NAS) to optimize the model accuracy, size, and training speed. In this study, the EfficientNetV2‐B0 model was used as the network, and fine‐tuning was performed using a model that had been pre‐trained with the ImageNet21k dataset. The number of epochs was set to 50, and the batch size was set to 32 for training. Adam was used as the optimization algorithm (optimizer), and dropout was set to 0.3. We employed early stopping to prevent overfitting. Automatic termination was performed when the validation loss did not improve by more than 0.001 for five consecutive epochs. These analyses were performed using an NVIDIA DGX Station A100. Finally, overall accuracy was used for evaluation.

## 3 RESULTS

### 3.1 Classification of native species and HYB

**FIGURE 3:**
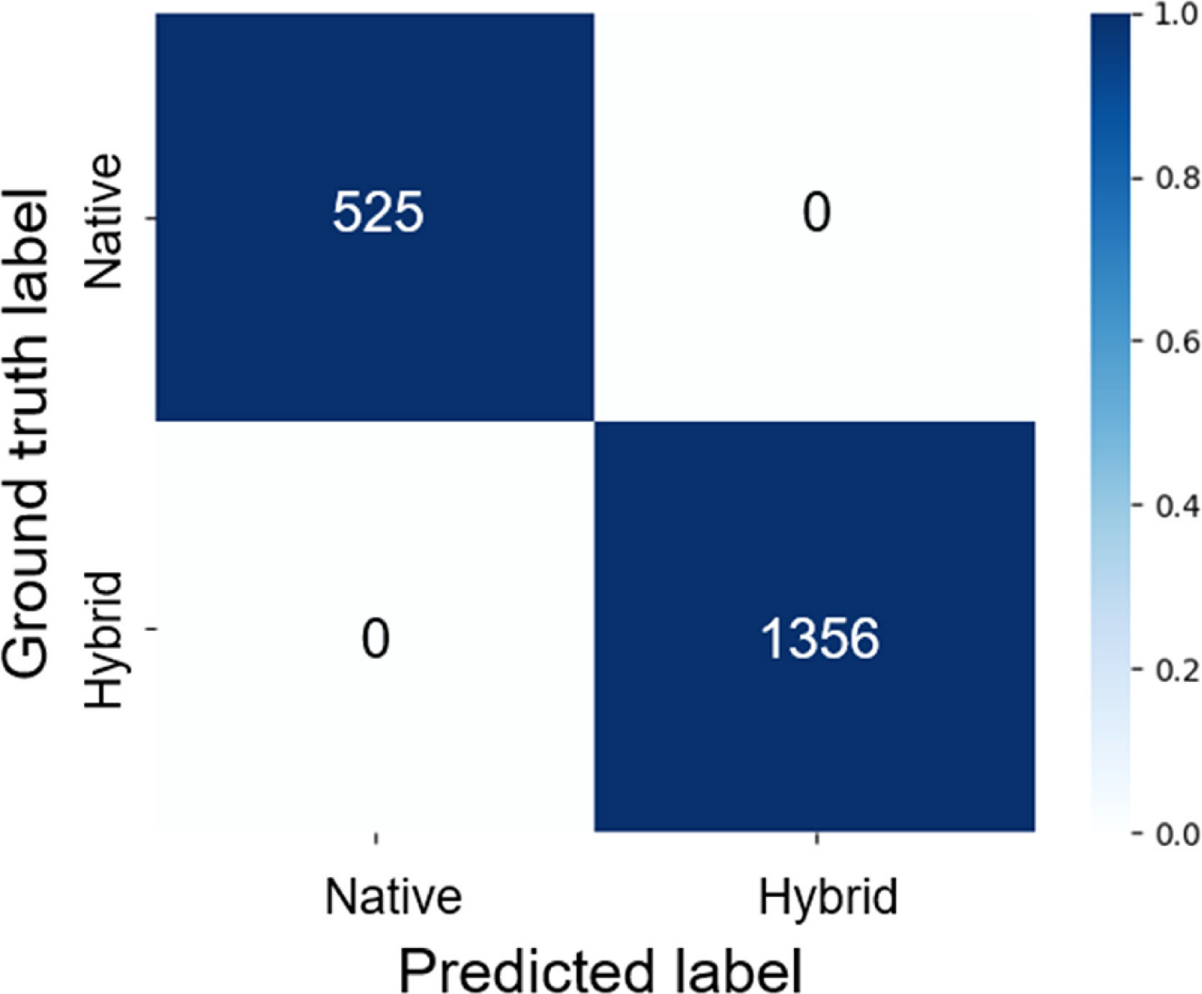
Binary classification of the native *Andrias japonicus* and HYB.

The six individuals of *A. japonicus* and 15 HYB used in the test (1881 images) were all correctly classified, namely with an accuracy of 100%, in our experimental environment. The classification results are presented in a confusion matrix (Figure 3), where the vertical axis is the ground truth and the horizontal axis is the model's prediction. The number in each cell indicates the number of images classified as native species or HYB, and the color of each cell indicates the percentage of images per ground truth. For example, pale blue indicated a ratio of 0.0, meaning that no images were classified for that cell. In contrast, dark blue indicated a ratio of 1.0, meaning that all images were classified for that cell.

### 3.2 Visualization using Grad‐CAM

**FIGURE 4:**
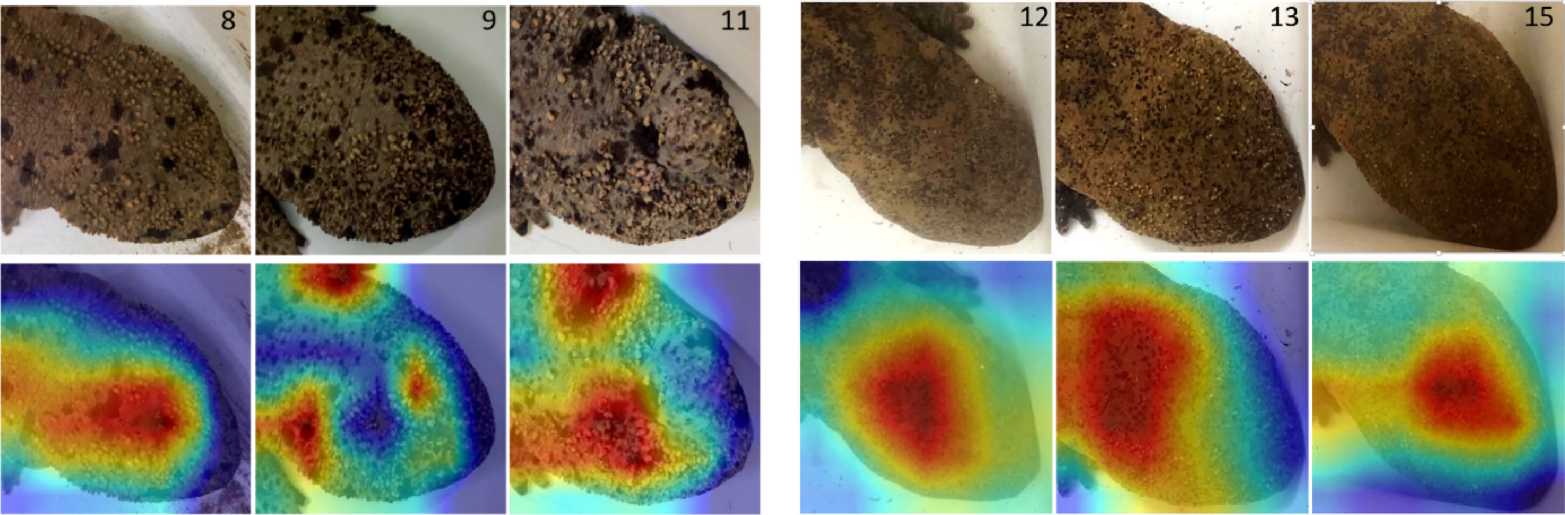
Visualization images generated by gradient‐weighted class activation mapping (Grad‐CAM). The three individuals on the left (8, 9, 11) were *Andrias japonicus*. Heatmaps indicate that the network learned the relatively distinctive large black spots of *A. japonicus*. The three individuals on the right (12, 13, 15) were HYB. In the case of HYB, the network learned the pale and ambiguous wide region.

The red regions are those considered by the model when outputting the prediction results (Figure 4 and Figures S4 and S5). The dorsal head spot patterning was a key area for classifying *A. japonicus* and HYB. The visualizations for *A. japonicus* indicate that the network learned to recognize the relatively distinctive large black spots. On the contrary, the visualizations for the HYB indicate that the network learned to focus on the pale and ambiguous wide region rather than the black spots. However, because the dorsal head spot patterning differed individually, the results of Grad‐CAM visualization also varied among them (Figures S4 and S5).

## 4 DISCUSSION

We identified HYB from images using deep learning. Historically, visual screening by experts and DNA analysis have been applied to identify HYB. However, the scarcity of experts and the time and cost of DNA analysis have been barriers to effective screening. Therefore, we proposed a novel approach to identifying HYB that uses an image recognition technique. A total of six native *A. japonicus* and 15 HYB individuals were used, and all were correctly classified by the AI model, namely the accuracy was 100%, in our experimental setting. Furthermore, highlighted regions that affect the AI model's prediction suggested that the model distinguished between native *A. japonicus* and HYB on the basis of spot patterns. Although deep learning has already been applied to identify species and individuals, to our knowledge, this is the first study in which it is used to identify hybrid individuals.

EfficientNetV2 demonstrated that dorsal head spot patterning can be used to identify *A. japonicus* and HYB. One reason why all individuals were successfully classified was the quality of the training and test images. In this study, photographs were taken from a short distance; thus, the high accuracy can be attributed to the clear spot patterns in the images. Another reason is that the heads were photographed from a similar angle (Figure S3). For example, previous studies have demonstrated that taking photographs from different angles reduces identification accuracy (Arzoumanian et al., 2005). Instead, we photographed all individuals from directly above the dorsal head and used them for training and testing images. Training and test images were also obtained on the same day, which could also have contributed to the high performance. In the future, the performance of our approach should be carefully evaluated in a varied environment, using images from different dates and locations, before it is implemented in the field.

The visualized distribution of the heatmaps was different for *A. japonicus* and HYB. For the former, the model focused on their distinctive large black spots, whereas for latter, it focused on the pale and ambiguous wide region. These results suggest that the differing spot patterns between *A. japonicus* and HYB can be utilized for classification. In general, *A. japonicus* have such black spots (Figure S1), whereas the spots of HYB are more indistinct (Figure S2). Experts use these spot pattern differences as one of the criteria to identify HYB individuals. Our study revealed that deep learning distinguishes between *A. japonicus* and HYB using the same pattern recognition as experts. The heatmap could be used as an instruction guide for the general public on HYB identification because the highlighted graphical figures are visually comprehensible. However, we could not analyze *A*. cf. *davidianus* because they are rarely found in Japan. Therefore, the dorsal head spot patterning of *A*. cf. *davidianus* should also be analyzed in cooperation with Chinese research institutions.

Although our approach achieved high accuracy in identifying *A. japonicus* and HYB in this study, several challenges still exist. Firstly, we did not consider the hybridization degree, which affects the spot pattern in HYB. The HYB used in this study were first generation (Shimizu et al., 2022), suggesting that these individuals have intermediate traits between *A. japonicus* and *A*. cf. *davidianus*. Since the HYB is fertile, the dorsal head spot pattern will depend on several factors, such as the generation. Future work should examine the relationship between the degree of genetic introgression and the identification accuracy. Secondly, it is essential to combine this method with DNA analysis because deep learning‐based identification has limitations. For example, due to hybridization, some HYB have spots indistinguishable from those of *A. japonicus*. DNA analysis is the only suitable method to determine the species in such cases. However, our technology can be applied for the early detection of suspected HYB through citizen science and rapid identification by computer vision. In addition, further advances in deep learning might enable the identification of backcrossed hybrids that are difficult to distinguish even for experts because their spots are extremely close to those of *A. japonicus*. Thirdly, the sample size was small because only five individuals were used for training. Therefore, greater accuracy can be expected by using additional training datasets. However, all HYB could be identified even when the sample size was small, suggesting that image recognition is an effective approach to detecting HYB. Finally, this study was conducted in the daytime under uniform photographic conditions, whereas *A. japonicus* and HYB must be photographed under artificial light in field surveys because they are nocturnal. In the future, it is necessary to determine whether images obtained under various light conditions can be used to identify HYB.

Hybridization between native and non‐native species is one of the major causes of biodiversity loss (Bourret et al., 2022). Moreover, hybrid individual detection is challenging when they are similar to the native species. Deep learning image recognition techniques can be a valuable tool to support the visual identification of hybrids. We proposed a new approach for classifying *A. japonicus* and HYB using smartphone images that could be utilized in citizen science. The artificial intelligence employed in this approach identifies HYB on the basis of spot patterns, a technique previously limited to experts, thus allowing the public to detect HYB easily. In particular, the distribution of HYB is expanding, meaning that managing them is a priority task for the conservation of *A. japonicus*. Our findings can potentially prevent their future spread by providing a method for the efficient discovery of such individuals. For example, citizens can find *A. japonicus* or HYB in the field and upload photos to social media such as X (Twitter), and our technology can facilitate the utilization of such online images.

## 5 CONCLUSION

We applied deep learning to identify *A. japonicus* and HYB. It was successfully demonstrated that the dorsal head patterning is an effective region for classification by Grad‐CAM. Although the visual identification of HYB has historically been restricted to specialists, our approach enables the public to identify them, making it particularly useful within the context of citizen science.

## AUTHOR CONTRIBUTIONS

**Kosuke Takaya:** Conceptualization (lead); methodology (lead); writing – original draft (lead); writing – review and editing (lead). **Takeshi Ise:** Conceptualization (supporting); methodology (supporting); writing – review and editing (supporting). **Yuki Taguchi:** Conceptualization (supporting); methodology (supporting); writing – review and editing (supporting).

## CONFLICT OF INTEREST STATEMENT

The authors declare no conflicts of interest associated with this manuscript.

## Supporting information


Figure S1

Figure S2

Figure S3

Figure S4

Figure S5

## Data Availability

The code and models are both also archived at Zenodo.
