# Supplementary material for: Identification of hybrids between the Japanese giant salamander (Andrias japonicus) and Chinese giant salamander (Andrias cf. davidianus) using deep learning and smartphone images
Source: Ecol Evol. 2023 Nov 9;13(11):e10698. doi: 10.1002/ece3.10698 (PMC10632944; doi:10.1002/ece3.10698)
Supplement: Supplementary file 1 — Figure S1 [file ECE3-13-e10698-s003.docx]

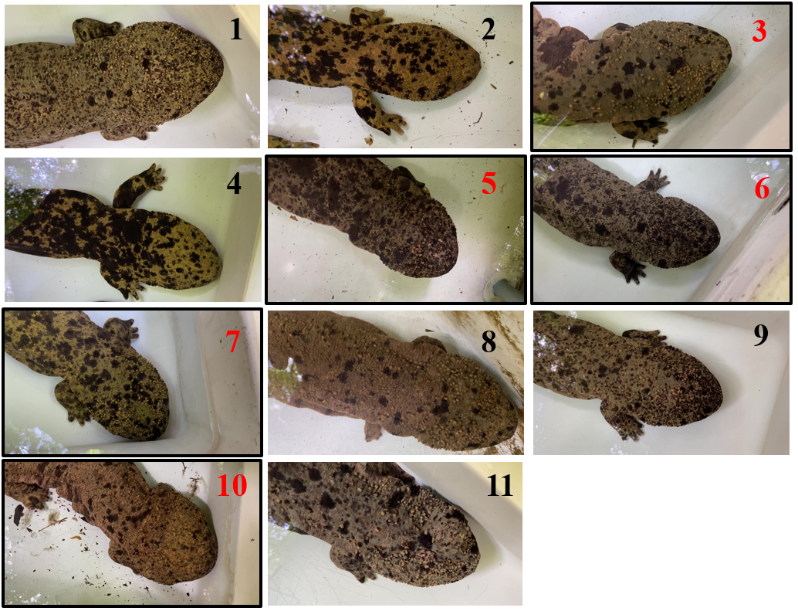


Supplementary Figure 1: Eleven individuals of *A. japonicus*. Individuals marked with red and black numbers were used for training and testing, respectively.
