# Supplementary material for: Identification of hybrids between the Japanese giant salamander (Andrias japonicus) and Chinese giant salamander (Andrias cf. davidianus) using deep learning and smartphone images
Source: Ecol Evol. 2023 Nov 9;13(11):e10698. doi: 10.1002/ece3.10698 (PMC10632944; doi:10.1002/ece3.10698)
Supplement: Supplementary file 3 — Figure S3 [file ECE3-13-e10698-s002.docx]

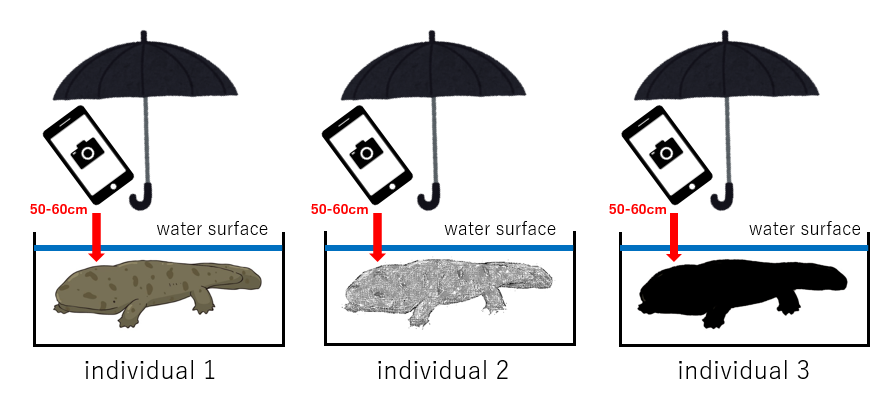


Supplementary Figure 3: Method of photographing *A. japonicus* and HYB. Photographs of underwater individuals were taken under a black umbrella to reduce reflection on the water surface.
