# Supplementary material for: Identification of hybrids between the Japanese giant salamander (Andrias japonicus) and Chinese giant salamander (Andrias cf. davidianus) using deep learning and smartphone images
Source: Ecol Evol. 2023 Nov 9;13(11):e10698. doi: 10.1002/ece3.10698 (PMC10632944; doi:10.1002/ece3.10698)
Supplement: Supplementary file 5 — Figure S5 [file ECE3-13-e10698-s005.docx]

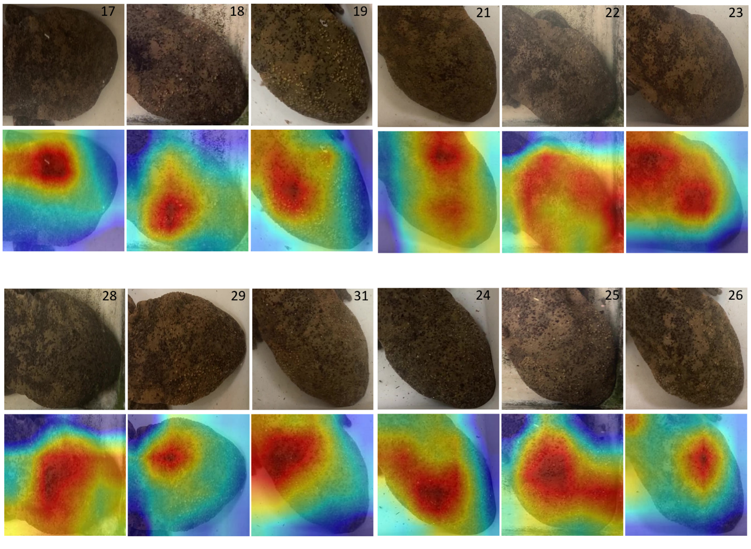


Supplementary Figure 5: Visualization of HYB individuals obtained using Grad-CAM on the trained model. The individuals depicted are different from those in Figure 4. The top row shows original images, and the bottom row shows heatmaps generated using Grad-CAM.
